# Exploring the experiences of college students in Chinese campus lockdown policy during the COVID-19 outbreak: A qualitative study

**DOI:** 10.1038/s41598-023-47182-w

**Published:** 2023-11-14

**Authors:** Zhiwei Li, Jinhui Lai, Caiyun Qi

**Affiliations:** 1 Department of Sociology, Shandong University, Jinan, Shandong Province China; 2 Department of Social Work and Social Management, School of Law, Shandong Jianzhu University, Jinan, Shandong Province China; 3 Department of Labor and Social Security, Jilin University, Changchun, Jilin Province China; 4 Department of Social Work, Shandong University, Jinan, Shandong Province China

**Keywords:** Psychology and behaviour, Public health

## Abstract

Campus lockdown policy is one of the most effective non-pharmaceutical intervention strategies used to prevent and control the coronavirus disease 2019epidemic worldwide. College students were greatly affected by this policy. Related studies center on English-speaking countries; few have highlighted discussion of the Chinese context. This study, therefore, aimed to broadly elicit the real experiences and unique insights of college students on Chinese campus lockdown policy. Through qualitative research, we identified four key themes with ten contributory subthemes: physiological experiences, safety experiences, love and belonging experiences, and self-esteem experiences. The unique contribution of this study relates to experiences relating to love and belonging and to self-esteem, which are little discussed in the existing literature. Our findings can provide enlightenment on how to improve college students’ health.

## Introduction

The coronavirus disease 2019 (COVID-19) is a global public health emergency^1^. It was declared a pandemic by the World Health Organization (WHO) due to its severity, which had infected approximately 63.64 million people across the world and caused 6.61 million deaths as of November 25, 2022^2^. The epidemic of coronavirus infections has posed an unprecedented risk to human life, health, and well-being. It has seriously impacted Chinese college students. According to data from the Ministry of Education in China^3^, about 44.3 million students in higher education in the country have been greatly affected by the COVID-19 epidemic. Particularly alarming is the fact that college students are more at risk of exposure to COVID-19 cluster outbreaks because of the living conditions of Chinese universities, like collective dormitory accommodation on campus and spaces of high-density activity^4^.

The campus lockdown policy (CLP) played a crucial role in reducing the risk of COVID-19 cluster infection among students. CLP usually meant closing a campus and letting all students study at home^5^. It emphasized that students should regard their home as an alternative space of education, thus ensuring the continuity of their education during COVID-19. According to empirical research, CLP could reduce peak incidence by 40–60 percent and delay the epidemic^6^. Therefore, most countries, such as the United States and Australia, implemented CLP extensively^7,8^. Colleges in China also adopted CLP. However, conditions were harsher than in other countries, with very different consequences that demand attention. In Chinese colleges in medium- and high-risk areas, off-campus students were ejected from campus and requested to continue their studies at home. For the larger proportion of students who lived on campus, a comprehensive and strict control strategy was implemented. This part of Chinese campus lockdown policy (CCLP) has played a critical role in reducing the risk of COVID-19 infection among college students. It involves: (a) security measures, such as complete lockdown in dormitories (*fengqin*), the prohibition of indoor gatherings, and the closure of all public places (e.g., libraries, gyms, classrooms) on campus; (b) emergency supply measures, such as door-to-door food delivery services, and distribution of daily necessities (e.g., toothpaste, drinking water); (c) educational guarantee measures, that is, comprehensive implementation of online teaching; (d) punishment measures, such as notices of criticism, warnings, or expulsion depending on the severity of the policy violation^9^.

Although most studies in other countries have reported increasing evidence of the effectiveness of CLP^10–12^, scholars have also found that implementing the policy had many negative effects on students. College students suffered physical shocks, including sleep disorders^13,14^, the impact of poor dietary habits^15,16^, and increased risk of cardiovascular disease^17^. In addition, several studies have highlighted psychological shocks experienced by college students, such as worsening depressive symptoms and extremely severe anxiety and stress symptoms^18–20^. Moreover, anxiety about academic performance during lockdown was caused by the challenges of online teaching^21,22^. The CCLP posed great challenges to Chinese college students. Bi et al. asserted that 69.0 percent of male and 73.5 percent of female college students in China suffered poor sleep because of the COVID-19 lockdown policy^23^. Hu et al. further found that college students’ lung capacity, endurance, and strength quality all declined^24^, putting their health at risk. Li et al. stated that the lockdown measures caused psychological problems among college students, including stress, anxiety, and depression^25^. The imposition of online learning during the long lockdown significantly weakened academic performance and freshmen and sophomores^26^. Evidence shows that many of these negative effects on college students were produced by feelings of hopelessness and deprivation arising from COVID-19 prevention and control measures^27,28^.

Our literature review found that existing studies mainly discuss the impacts of COVID-19 measures on students’ physiological and psychological conditions. However, due to the strict nature of CCLP, the challenges faced by Chinese college students quarantined in on-campus dormitories were much more severe and pervasive. In our informal observation, we found that the implementation of CCLP not only caused physical and psychological health problems among college students, but also significantly impacted their social activities, romantic relationships, personal freedom, and so on. News reports and social media messages showed that some college students even committed suicide during the lockdown^29,30^. However, little attention has been paid to the huge adverse consequences of CCLP. Chinese colleges will likely continue to adopt CCLP in response to similar epidemics in the future, so it is necessary to understand the actual experiences of Chinese on-campus college students to reduce the negative effects of this policy.

In the current context, the experiences of CCLP are highly correlated with the inability to meet various aspects of human needs. We need to conceptualize these needs through related needs theory. Among them, Maslow's hierarchy of needs model is the most widely known. Abraham Maslow’s 1943 seminal work, “A Theory of Human Motivation,” provides a potential framework for a holistic consideration of people’s needs^31^. He believed that as an inherent aspect of human existence, there are not only physical needs, but also psychological needs. The environment must well satisfy these needs, otherwise, physical and mental illness will occur^31^. In this regard, he put forward the hierarchy of needs model from the human motivation approach, emphasizing that human motivation is determined by human needs. This model divided the fundamental needs of human beings into five levels from low to high: physiological needs, safety, love and belonging, self-esteem, and self-actualization. The first four are often referred to as deficiency needs, which directly endanger people’s life when they are not met. The last is the growth need, which is not absolutely necessary for the survival of the individual, but satisfying this need makes people healthy, longevous and energetic^32^. This model creatively classifies people's diverse needs and points out the significance of different needs for individual survival and development^31^. It offers a common analytical framework for understanding and action rather than a rigid prescription governing all human activity^33^.

Although Maslow's hierarchy of needs is widely used, we still need to review different opinions and criticisms to make the framework more effective. The main query comes from the reality of the hierarchy. Some studies believed that people still have higher-order needs even if their rudimentary ones are not met^34^. Some scholars have also proposed that the model is too simplistic and fails to take into account cross-cultural effectiveness^35^. Tay and Diener (2011) tested Maslow's theory by analyzing data from 60,865 participants from 123 countries and found that Maslow's ranking of needs was incorrect, but the prevalence of the types of needs proposed was verified in different countries^36^. Our review of different needs models further proves that these models have different attitudes towards the relationship between different types of needs, but they use Maslow's classification to a great degree^37–39^. For example, through empirical studies in the United States and China, Nevis divided the hierarchy of needs theory into four levels: belonging (social) needs, physiological needs, safety needs, and self-realization needs^40^. The double-Y model of basic human needs proposed by Yang pointed out that on the left arm of the Y are interpersonal and belongingness needs, esteem needs, and self-actualization needs. Y's right arm are sexual needs, childbearing needs, and parenting needs^41^. Alderfer's need theory folds Maslow's five types of needs into three: survival needs (air, money, and hunger), relation needs (affiliation and friendship), and growth needs (to be creative and to reach the full potential)^42,43^.

Therefore, combining Maslow's need model, other need models, and related criticism of Maslow's need model, this paper does not adopt the hierarchy, but only uses the classification, and classify college students' negative experience into four types. In the CCLP context, the main contents are as follows: First, physiological experience is defined as a personal physiological response to objective conditions, including air, water, food, shelter, sleep, homeostasis, etc. Second, safety experiences. When people felt threatened by things around them and that the world was dangerous, they felt insecure in their hearts. In this element, we build upon the original basic human need for safety from physical and psychological threats to include the contemporary issues of personal and financial security. Third, love and belonging experiences. Friendly and intimate relationships and identifying with a particular group or groups are essential human needs. This factor contained two meanings: on the one hand, it involved maintaining good emotional relationships with friends or partners and obtaining support from each other. On the other hand, they were eager to maintain good social relations with others and to be accepted into a social group. Last, self-esteem experiences. Respect affirms an individual’s abilities, qualities, or identity. A positive, respectful environment nurtures both wellness and productivity. This element included not only an internal respect experience relating to self-recognition and to being independent and free, but also an external respect experience relating to the desire to be recognized and treated fairly by others^44,45^. It is worth noting that "self-realization" is a growth need. In our research, the satisfaction of deficiency needs is more prominent. After repeated discussions by researchers, it was not included in this study.

By interviewing 21 Chinese college students, this study scrutinized the negative experiences and feelings of Chinese college students in on-campus isolation. The remainder of this paper is organized as follows. Section 2 introduces the details of the method and materials. Findings are presented in Sect. 3, followed by discussion of the key findings in Sect. 4. The paper ends with discussion of limitations and a brief conclusion. Our study addresses the critical research gap.

## Methods

### Research design

We turned to John W. Creswell’s *Qualitative Inquiry and Research Design* to guide this study’s design. Specifically, we used the method of hermeneutic phenomenology, which allows the interrogator to collect data from people who have experienced a specific phenomenon and to develop a composite description of the essence of that experience for all those individuals^46^. This description consists of “what” they experienced and “how” they experienced it, which helps explore the deep insights of college students on CCLP. Data were collected from individuals who had experienced CCLP through semi-structured interviews. The raw data were processed by content analysis. The results of this study carried out the consolidated criteria for reporting qualitative Studies (COREQ) guidelines^47^.

### Sample and recruitment

We selected Beijing, Shanghai, and Changchun as research sites. These three regions have been hit hardest by the epidemic. Moreover, they are located in different parts of China, with different economic levels and living environments, which helped us to collect as much data as possible. Recruitment of potential participants occurred through advertising on social media (e.g., campus post bar, Zhihu, Xiaohongshu) and recommendations from acquaintances.

Twenty-one participants (10 female, 11 male) were selected by purposive sampling^48^. The inclusion criteria were as follows: participants had to (a) be full-time college students who had experienced campus lockdown (“fengqin”) for more than one month, (b) have lived on campus during campus lockdown, and (c) be willing to take part in the study. To construct comprehensive understanding, we used the maximum variation strategy to balance the sample according to the respondents’ characteristics^49^ such as age, gender, educational attainment (bachelor, master, and doctorate candidates), academic discipline (social sciences, human sciences, and natural sciences), and university location (Beijing, Shanghai, and Changchun). The study’s sample size was determined by data saturation. Saturation means that no new information or themes are found in the empirical data, indicating that data collection may be complete^50^. To assess data saturation, we used the criterion of Hennin, Kaiser, and Marconi^51^. First, we documented the process of code development according to the interview sequence. Second, we classified and analyzed the coding. Last, when we interviewed the 21st participant, we found that the same code reappeared and we did not obtain a new theme. In order to confirm the data saturation, we recruited more three participants for interview, but no new themes or information appeared. We determined that the data was now saturated. We only analyzed the data from the first 21 interviewees.

The frequency descriptions of the sociodemographic characteristics of the 21 participants are shown in Table 1. The number of men (*n* = 11; 52%) and women (*n* = 10; 48%) was nearly equal. Age statistics show that most participants were between 20 and 25 years old (*n* = 13; 65%), average age 23.8. The educational characteristics of the participants indicate that most were undergraduates (*n* = 12; 57%), and the social sciences had the largest number of participants (*n* = 13; 61%). The numbers of participants from universities in Beijing (*n* = 6; 29%), Shanghai (*n* = 7; 33%), and Changchun (*n* = 8; 38%) were basically the same.

**Table 1:** Characteristics of participants (*n* = 21).

Characteristics	*N* (%)
Age (years)	
20–25 (included 25)	14 (67)
> 25	7 (33)
Gender	
Female	10 (48)
Male	11 (52)
Educational attainment	
Bachelor	12 (57)
Master	7 (23)
Doctor	2 (10)
Academic discipline	
Social Sciences	13 (61)
Human Sciences	2 (10)
Natural Science	6 (29)
City	
Beijing	6 (29)
Shanghai	7 (33)
Changchun	8 (38)

### Data collection

We collected data through one-to-one semi-structured interviews held between March 1, 2022, and June 28, 2022. Compared to focus-group interviews, one-on-one interviews are conducted in a more private setting, facilitating the expression of introverted participants or sensitive topics. All interviews were conducted online using Tencent Conferencing software, an online communications platform used in audio and video conferences, classes, and interviews during the COVID-19 pandemic. We started by asking the participants two broad, open-ended questions: What did you experience in terms of the CCLP? What contexts or situations typically influenced or affected your experiences of the CCLP? In addition, in order to collect richer information, we designed an interview guide based on the research purpose and literature review. It was then modified by two pilot interviews and reworked in line with experts’ advice to ensure that the final interview guide was easy to understand (see Table 2). We also reiterated the purpose and significance of the study to the participants and asked for written consent and permission for the interviews to be recorded. During the interviews, the researchers listened carefully, remained neutral, encouraged the participants to express their ideas fully, and recorded important information efficiently. After each interview, we recorded a reflection diary the same day. All of our interviews were conducted in Mandarin Chinese, and each lasted approximately 30–60 min.

**Table 2:** Interview guide.

1. What did you experience in terms of the CCLP?	
2. What contexts or situations typically influenced or affected your experiences of the CCLP?	
3. Was your physiological state affected during the campus lockdown?	
4. Do you think your safety was at risk under the CCLP implementation?	
5. Did you notice any changes in your social contacts during the campus lockdown?	
6. Was your self-esteem affected during the campus lockdown?	
7. What do you think are the shortcomings of CCLP? Do you have any suggestions for its improvement?	
8. Is there anything missing (or to add) about the topic we’re talking about today?	

### Data analysis

All interviews were recorded and transcribed word-for-word into Mardarin Chinese by the first author (ZL) and second author (JL), and some information was translated into English selectively by the first author, as needed to present the research results. The third author (CQ) checked the Chinese transcripts and translations several times to ensure accuracy. These materials were repeatedly read and reflected on by all authors and coded independently. Then, we discussed the data analysis and coding processes in detail, to ensure that the value of the data and the rationality of the coding were maximized. Organization and analysis of the data were assisted by Nvivo 11 software (see Supporting Information for details).

The purpose of this research is to present the voices of Chinese college students and explore their needs and experiences for CCLP during the COVID-19 epidemic. Their experiences are complex and diverse. We draw on the classification by Maslow's need model, other need models, and related criticism of Maslow's need model, to make the data collection and analysis processes clearer^50,52^. We used a qualitative content analysis method^48,53^. ﻿Content analysis is a research method for making replicable and valid inferences from data to their context, with the purpose of providing knowledge, new insights, a representation of facts and a practical guide to action^54^. It is usually classified as primarily a qualitative versus quantitative research method^55^. Qualitative content analysis goes beyond merely counting words to examining language intensely for the purpose of classifying large amounts of text into an efficient number of categories that represent similar meanings. The goal is “to provide knowledge and understanding of the phenomenon under study”^53,56^. Specific steps in this study as follows: (a) the unit of analysis was selected, and the transcript sentences reflecting Chinese college students’ negative feelings about CCLP were classified into much smaller content categories to form the unit of analysis; (b) the original data were reviewed and read through several times; (c) the classification outline was designed based on the research framework to identify the categories of units of analysis; (d) content coding and classification were carried out; the essential contents and concepts in the data were coded and marked openly, and similar codes were grouped into corresponding categories to present themes and sub-themes; (e) the findings were explained and analyzed, the data and findings were finally linked, and related data examples were identified.

### Trustworthiness

In this research, to ensure the trustworthiness of the interview data, we applied four criteria specified by Lincoln and Guba^57^: (a) to increase the credibility of the study, we ensured that every researcher had received rigorous academic training in areas including reflection diaries, completion of interview notes following standardized procedures, etc., and that peer reporting was conducted with colleagues in the department; (b) to ensure the standard of transferability, we described the interview participants in detail and comprehensively analyzed the research process; (c) transcripts were coded independently by all researchers to ensure that the value of the data was developed maximally; (d) we adequately cited the participants' statements on the themes, and each reference had an independent code to ensure confirmation of the results.

### Ethics

The study was conducted in accordance with the Declaration of Helsinki, and approved by the Academic Committee of the School of Law, Shandong Jianzhu University (2022-04-06). All participants provided their written informed consent to participate in this study. We provided participants with relevant information, including the topic, purpose, duration, and research schedule. More evidence details are documented in the interview recordings and transcripts.

## Results

Due to the open nature of the interviews and the expected purpose of improving policy to provide reference information, we focused on analyzing all responses to negative experiences. Following detailed content analysis with interview transcripts, we identified four themes and ten contributory subthemes (see Table 3). The quotations displayed below in discussion of each theme were carefully selected to present the most important emotions and feelings experienced by the participants.

**Table 3:** Themes and sub-themes.

Themes	Sub-themes
Physiological experiences	Food challenges
	Sleep disorder
	Shelter barriers
Safety experiences	Physical insecurity
	Psychological insecurity
	Financial insecurity
Love and belonging experiences	Obstacles to group gatherings
	Obstacles to maintaining relationships
Self-esteem experiences	Being controlled
	Not being respected

### Theme 1: Physiological experiences

The most frequently mentioned factors were physiological, relating to what participants described as food challenges, sleep disorder, and shelter barriers.

#### Food challenges

The difficulty of meeting daily food needs was the most common negative response reported by participants. Most said that their schools’ unified distribution of meals had been relatively fixed for a long time, and hygiene was not guaranteed, which caused dissatisfaction. As Participant 6 said, *“School catering dishes have been single-kind long-term, and there was an event of group diarrhea, causing many students to complain”* (Participant 6, aged 20, female, bachelor)*.* Before the implementation of CCLP, college students would choose to buy meals through various channels (e.g., takeout platforms and campus canteen halls). However, after implementation, they were quarantined in the campus dormitory. Obtaining their preferred foods became a challenge; they lost the right to choose their food. Participant 2 described her experience: *“Girls don’t like meat so much but need to eat vegetables more. When the school arranges food uniformly, there is no choice”* (Participant 2, aged 26, female, master)*.* In short, faced with the dual distress of the school’s unified meals not meeting their needs and being deprived of choice, the participants’ experiences became worse and worse. Furthermore, many students ate little or no food under these circumstances, leading to food waste:

*Just think, we ate (unified meals arranged by the school) for more than a month. I wanted to throw up. Many students didn’t eat it and threw it away, resulting in a great waste of food. (Participant 1, aged 26, female, master)*

Many participants reported that the fruit and food supplies on campus were also impacted by the CCLP implementation. Participants described that to reduce people-to-people contact, schools developed online smart mall shopping platforms to sell daily necessities. However, for some participants, shopping in the smart mall was also a big challenge due to the shortage of goods, especially fruits and snacks. Participant 5 complained:

*During the lockdown period, in order to rush to purchase the fruit and instant noodles, I even set the alarm clock, watching all the time, but it was no use. Too panicky. Buying is all by luck! The goods are far too scarce! I think it’s burdensome! (Participant 5, aged 21, female, bachelor)*

#### Sleep disorder

Unexpectedly, most of the participants described being troubled by sleep disorders during lockdown. It should be noted that they reported sleeping well before the policy was implemented. However, a while after implementation, they had difficulty falling asleep or woke easily and repeatedly. This led to listlessness, affecting their studies and life. Participants thought the long-term lockdown was a critical factor that caused psychological discomfort and even long-term fantasies of sick psychology, resulting in the deterioration of sleep quality:

*I was very anxious during the lockdown. I was always thinking wildly. I couldn’t sleep at night, and I stayed up late, so I was late for the next day’s online classes. (Participant 21, aged 22, female, bachelor)*

#### Shelter barriers

Participants reported that the distress and troubles caused by the poor dormitory environment had increased during the CCLP implementation. They had long been confined to the collective dormitory, with four to six students living together in an area of only a little over ten square meters. Interviewees described feeling a strong sense of space pressure, aggravating their discomfort with their accommodation:

*The layout of our dormitory is very unreasonable, with dim light and limited space. Usually, I don’t like to stay in the dormitory. At that time, being blocked in the dormitory for a long time, I often had a false sense of time and space and felt very depressed. (Participant 18, aged 27, female, doctor)*

### Theme 2: Safety experiences

Although the implementation of CCLP reduced the risk of COVID-19 infection among college students, it might have threatened their health and finances. These threats were primarily manifested in physical, psychological, and financial insecurity.

#### Physical insecurity

During lockdown, most interviewees said that they faced tremendous challenges in terms of physical health. The long process of buying medicine and the cumbersome procedures were the most frequently mentioned challenges. Participant 5 described her feelings: *“My roommate had a mouth ulcer. She needed to go through a tedious application process to buy medicine. It took several days to get the medicine. She was in pain for days”* (Participant 5, aged 21, female, bachelor)*.* Other participants said that the difficulty of seeing a doctor offline was a big problem. Participant 15 described his experience: *“I had an ear infection, and I could only get a consultation online, but that is not as good as seeing the doctor in a clinic offline. I was still waiting for the school to be freed from lockdown to go to the hospital for further examination. I could only pray”* (Participant 15, aged 21, male, bachelor)*.* It was difficult to buy medicine and to see a doctor offline during lockdown, which delayed treatment, aggravated conditions, and threatened their health.

#### Psychological insecurity

Psychological insecurity was a negative experience reported by almost all participants. First, they were worried and anxious about the effectiveness of online teaching. Compared with the traditional offline teaching mode, online teaching has many disadvantages, like poor concentration, hardware and software technical barriers, and a lack of emotional interaction between teachers and students. These disadvantages affected their learning results and produced insecurity about future learning outcomes:

*With online classes, it’s easier (for me) to get distracted, and I have less interaction with the instructor. Furthermore, when roommates had courses simultaneously, the internet was slow, and the course experience was not good. (I was) worried that the learning effect was not guaranteed. I was pretty anxious. (Participant 2, aged 26, female, master)*

Second, most participants worried about employment during long-term lockdown. On-campus students were barred from leaving their school, preventing them from attending outside job interviews. Although the Chinese government encouraged employers to recruit online, the jobs themselves were offline (e.g., civil servant positions, technical positions). If students couldn’t participate in interviews or exams in person or demonstrate practical skills on the spot, they had to give up job opportunities. Participant 5 described the job dilemma as follows:

*We lost many job opportunities because we had no way to go for interviews, nor could we go to other provinces to take the examinations administered by public institutions. I felt very anxious. (Participant 5, aged 21, female, bachelor)*

Last, many participants expressed that “the uncertainty of the unlocking time” was a key factor in their insecurities as well. They said this mainly stemmed from concerns about the effectiveness of the government in dealing with the epidemic. Participant 4 expressed his feelings:

*The COVID-19 epidemic in Shanghai was constantly recurring. The government always told us that the epidemic would be eliminated soon, and we followed the government’s orders very well. Sadly, we waited a long time for news of the unlocking policy from the government. I felt very disappointed. (Participant 4, aged 20, male, bachelor)*

#### Financial insecurity

During lockdown, most college students faced unexpectedly increased financial pressures. Due to the CCLP implementation, they were banned from leaving their school, and delivery services were prohibited. Thus, unscrupulous merchants on campus seized this opportunity to raise commodity prices. Participant 3 said:

*During the lockdown, the goods sold in the school store were much more expensive than usual. For example, the same box of Coke can be purchased from online shopping malls (e.g., Taobao, Jingdong), and the total cost is only about 40 yuan plus delivery. But the school store charged 82 yuan for a box. Everyone thinks the merchants on campus were making dirty money. They were getting rich. (Participant 3, aged 22, male, bachelor)*

Notably, some participants were concerned that lockdown had disrupted their income from part-time jobs. This was a serious blow for poor students who relied on part-time jobs to cover their living expenses. As Participant 20 described,

*My classmate is an art student, and her family’s economic situation is not very good. I know she regularly went to teach children painting in a preschool on Saturdays and Sundays. However, during the lockdown, part-time jobs were not possible. Thus, her income was interrupted. Furthermore, it meant she couldn’t support her basic life needs. (Participant 20, aged 22, female, bachelor)*

### Theme 3: Love and belonging experiences

Obstacles to group gatherings and maintaining relationships were the main negative love and belonging-related experiences of college students under CCLP. These negative factors posed great challenges to their social life and emotional state.were concerned that lockdown had disrupted their income

#### Obstacles to group gatherings

During the CCLP implementation, participants were forced to interrupt all offline social activities. Long-term social disruption can cause emotional disorders, such as diminished social awareness and regression of social skills. Participant 10 described her experience: *“After experiencing the long-term lockdown, I didn’t know how to maintain relationships with friends, and my circle of friends has become strange”* (Participant 10, aged 25, female, master). The ban on social gatherings is a crucial measure of CCLP. Under lockdown, students’ need for gatherings was not met, which may exacerbate negative feelings. Participant 13 describes this as below:

*The lockdown was too long, and I couldn’t have dinner with my friends. My heart was filled with anxiety. (Participant 13, aged 24, male, bachelor)*

Moreover, participants commented on offline social activities being suspended, and many bad habits were gradually developed, such as addiction to electronic devices (e.g., smartphones, tablets), staying up late, and laziness:

*After social activities were restricted, (I) found that the girls who were originally beautifully dressed every day began not to make the same effort. Their hair was not washed as often. (I) feel that they have become very lazy. (Participant 6, aged 20, female, bachelor)*

#### Obstacles to maintaining relationships

It was widely reported that lockdown management greatly impacted friendships among college students and that they were more prone to conflictual behaviors. For instance, small frictions in their lives could become magnified at this time and lead to violent arguments. This might have to do with the fact that they were quarantined long-term in a fixed environment and became depressed and sensitive. Participant 1 expressed her feelings:

*We were isolated in such a small space in the dormitory for a long time, and my roommates became sensitive to each other’s emotions. They often disliked each other because of small things, quickly causing conflicts. (Participant 1, aged 26, female, master)*

Participants also said that their relationships with friends outside of school became more distant. One of the primary reasons was that they had lost the experience of real emotional interaction with friends. Participant 13 describes the friendship dilemma he encountered:

*My relationships with friends outside of school have been weakened greatly because of the CCLP implementation, because we lost the opportunity for face-to-face emotional communication. (Participant 13, aged 24, male, bachelor)*

It is worth noting that romantic relationships were also affected by CCLP. Some participants described that because of lockdown, couples on campus could not see each other offline for a while, denying them intimacy. Many eventually broke up. Participant 18 reported that.

*I feel that it has had a huge impact on campus couples. After the CCLP was implemented, the two sides (of the couple) couldn’t meet for a long time … As far as I know, several couples have broken up. (Participant 18, aged 26, female, doctor)*

### Theme 4: Self-esteem experiences

Besides the negative experiences described above, participants also said their self-esteem was affected in the context of the CCLP implementation. This negative effect mainly included two factors: being controlled and not being respected.

#### Being controlled

Many participants were concerned that their freedom was severely constrained, being blocked in dormitories, prevented from leaving the school, and prohibited from public places such as libraries, gyms, and so on. Policymakers tried to restrict the freedom of college students legally and legitimately through public power, because restriction of liberty is one of the most effective non-pharmaceutical interventions in epidemic prevention and control^58^. However, many participants described CCLP as a form of “imprisonment.” Participant 13, for example: *“We were quarantined in the dormitory during the CCLP implementation … I felt like I was ‘in jail,’ I waited for the time when we could get out”* (Participant 13, aged 24, male, bachelor). In such a situation, students were likely to engage in rebellious behavior, as Participant 4’s report shows:

*During the lockdown, many students on campus couldn’t bear the restriction of their freedom for such a long time and secretly climbed over the wall to escape. In addition, I heard several students from the nearby university climbing over the fence and leaving the campus, causing cross-infection of COVID-19 among students. (Participant 4, aged 20, male, bachelor)*

#### Not being respected

As policy objects, participants deeply experienced the good and bad aspects of CCLP. The “bad” part (shortcoming) of the policy might affect the maximization of policy realization. Therefore, their suggestions and opinions concerning the policy are very valuable. However, many participants reported that policymakers ignored their suggestions. Participant 1 expressed her experience:

*The school provided us with free bottled water, but we didn’t need bottled water because there was a water fountain in our dormitory building. We told our teacher that we didn't need bottled water a few times, but the school kept delivering water to us. It felt so wasteful. (Participant 1, aged 26, female, master)*

In this case, participants felt that they were not respected. One described this experience: *“the school would not take my suggestion. My suggestions and needs were always ignored, and I felt disrespected”* (Participant 16, aged 25, female, master). This disrespect increased students’ incomprehension of the policy. For example, Participant 17 described the policy implementation process as *“very formalistic”* (Participant 17, aged 23, male, bachelor).

## Discussion

Using a qualitative research method, we investigated college students’ unique insights into and real experiences of CCLP during COVID-19. We found that they were affected in a range of ways by CCLP, especially regarding meal needs, physical and mental health, social emotions, personal freedom, and other significant challenges. Ultimately, we identified four themes in the adverse experiences caused by CCLP: physiological, safety, love and belonging, and self-esteem. The findings of this study should provide not only new perspectives for future research but also present policymakers with unrecognized negative effects of CLP implementation in the Chinese context.

First, we noted that college students had a strong negative response to their physiological experiences, including food-related problems, sleep disorders, and concerns with the dormitory environment. These findings support earlier studies that found effects on dietary habits^59^and severe sleep disorders^60^. In addition, we have shown that college students faced a greater challenge when quarantined in their campus dormitories. This may be related to the unique campus accommodation environment in China, especially its overcrowded living space.

Second, a unique theme highlighted in this study is that college students faced multiple threats to their safety, manifested as physical health challenges, psychological insecurity, and increased financial burden. Many western scholars point out that CLP is implemented to effectively control the spread of COVID-19 among college students^61,62^. However, college students’ physiology, psychology, and learning were impacted by the policy^19,63,64^. Our study also generally confirmed the results of prior studies. Most importantly, we conducted further categorization to help policymakers identify more clearly the negative effects caused by the policy. Notably, we added a previously unrecognized experience – increased financial burden, which led to a struggle to survive among vulnerable poor students.

Third, the unique contribution of this study is that we have presented the love and belonging and respect experiences of college students under CCLP, which are not discussed in the existing literature. Love and belonging experiences mainly included two negative factors: the interruption of social activities and the crisis of friendship and love. The college campus is a crucial platform for students to establish friendships and find love. However, unlike the CLP implemented in other countries, Chinese college students were confined to campus dormitories, and offline activities were banned. As a result, the social functions of college campuses were limited, which could lead to the crisis of broken social and emotional ties.

Finally, self-esteem experiences mainly manifest in the denial of personal freedom and refusal of the right to expression. It was found that the enforced “physical isolation” intended to protect and prolong the length of an individual’s life led to the freedom dilemma^65^. We have no intention here of exploring what the limits of freedom should be but only to present the reality that college students were excessively deprived of freedom under CCLP. Moreover, as the policy target, college students were excluded from the policymaking, and their suggestions and appeals were ignored.

**Figure 1:**
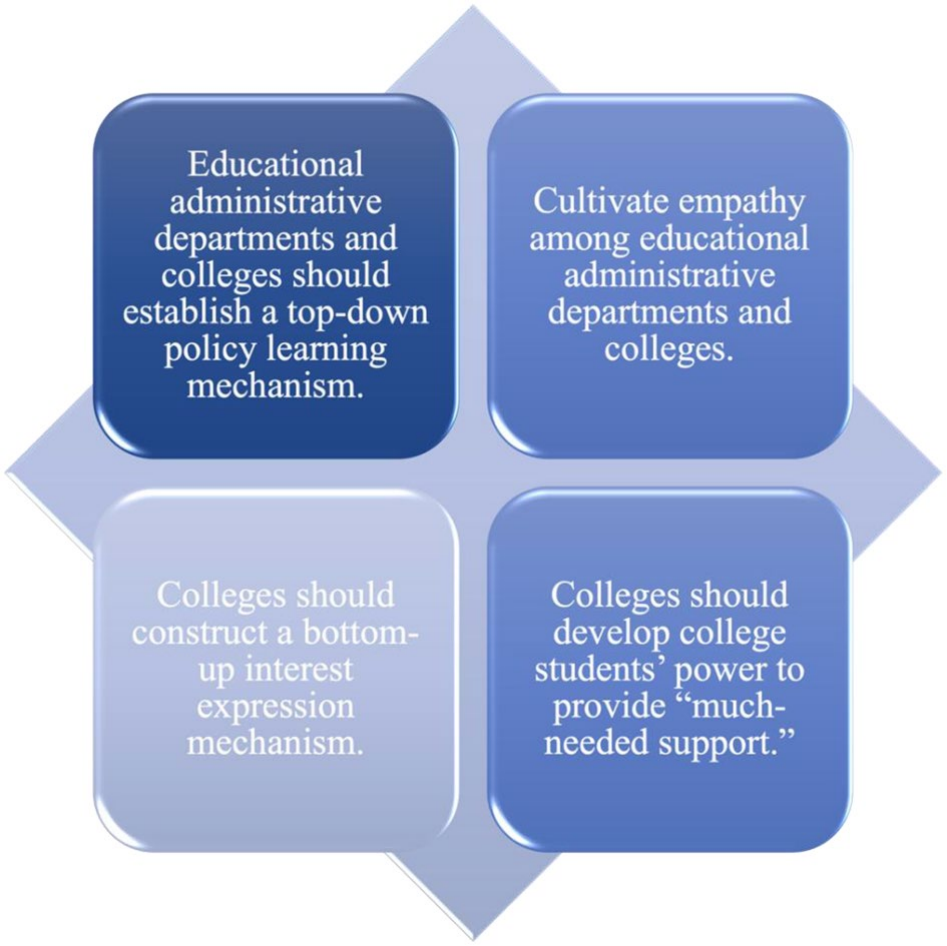
The suggestions for improving the CCLP loophole.

Chinese college students’ negative feedback on CCLP is closely related to the policy implementation mode under the authoritarian Chinese regime. In China, the government adopted a “zero-COVID policy”^66^, including a series of measures, such as city lockdown, the closing of public places (including schools), home quarantine, and suspension of all public transportation^67^. It is a top-down model of political mobilization, in which a strong central government pushes down tasks and relies on strict accountability to motivate lower-level governments^66^. This ensured that China achieved effective epidemic prevention and control^68^. However, top-down policies lack flexibility^69^, and policymakers lack empathy for policy objects^70^, which led to insufficient protection of college students’ human rights and a series of negative experiences. To address the policy loopholes, we introduce the following suggestions (see Fig. 1).

First, establish a top-down policy learning mechanism. We suggest that educational administrative departments and colleges extensively investigate and evaluate problems in the CCLP implementation at universities in Shanghai and Jilin, and adjust policies in a timely manner based on factual data to avoid the recurrence of the same problems.

Second, cultivate empathy among policymakers and implementers. In order to avoid a continuous negative cycle of tragedy, educational administrative departments and colleges should put themselves in the shoes of college students and take the initiative to eliminate policy loopholes. For example, healthy meals should be ensured through diversity and options. More appropriate accommodation should be created through the readjustment of the number of students in one dormitory. These measures are crucial for college students to enhance their immunity and resistance, reduce discomfort in the residential environment, improve sleep quality, and increase social interaction.

Third, construct a bottom-up interest expression mechanism. Colleges not only need to open channels for students to express their policy demands and suggestions, but also give timely feedback on their demands and formulate effective responses to their questions, to eliminate the sense of insecurity and disrespect.

Last, give college students the freedom to provide “much-needed support” for campus epidemic prevention and control. In this study, we deeply understood some college students’ spontaneous involvement in epidemic prevention and control, such as contributing spare medicine to help sick students and acting as close companions for students with psychological disorders. These seemingly small gestures show the advantages of grassroots mutual assistance in fighting the pandemic. It is necessary to strengthen the education of college students, and cultivate their ability to deal with crisis and the spirit of mutual help, to form an effective and powerful alliance to fight against epidemics.

Campus lockdown policies are one of the most effective non-drug intervention strategies for the prevention and control of COVID-19, but they also have many unwanted consequences. Therefore, we use Maslow's need model, other need models, and related criticism of Maslow's need model, to design need types, and explore the specific feedback of Chinese college students to CCLP. This model also provides a framework for intervention by the state, schools, and individuals. However, this does not mean that the lockdown policy on the campus is reasonable after meeting the needs. The stance towards lockdown policy is full of opposition and conflict, although the rationale for lockdown is well-sustained by strong epidemiological arguments. The counterargument is that it has other adverse consequences that are more serious, such as the use or abuse of human rights and freedom restrictions, economic issues, marginalized groups and eclipse of all other diseases^71,72^. Unmet individual needs are only one aspect. This paper does not explore the rationality of the campus lockdown policy itself. We, only under the established reality, according to the needs of college students, put forward ways to improve this policy. Further research should be carried out on the rationality of the CCLP in a more comprehensive manner, to avoid contributing to history with yet another mistakes, as seen in other past epidemics.

## Limitations

As far as we know, this is the first study to investigate CCLP. Through a standardized process, we collected much data about the experiences of 21 college students in Beijing, Shanghai, and Jilin and extracted findings. These findings provide unique empirical evidence for improving and optimizing CCLP. There are also two limitations: for one thing, as the participants were only recruited from Beijing, Shanghai, and Jilin, the generalizability of the findings to other regions needs further investigation. On the other hand, participants were interviewed at Tencent's conference. Network interview breaks the space limit and can achieve higher anonymity. Inevitably, however, the disadvantage is that the lack of extra-verbal information (facial expressions, body language, etc.) can produce ineffective response biases, and it is difficult to get a rich and detailed description. Although the interviewers in the research have been professionally trained, there is still a risk that they cannot better understand the interviewee's emotions and expressions through the body language. In the future, we should conduct more qualitative investigations to enhance our understanding of this issue.

## Conclusion

The CCLP plays a critical role in reducing the risk of COVID-19 infection among college students. However, the policy also posed students great challenges. This study has identified four negative experiences caused by CCLP. To improve this policy, we put forward some suggestions, including building a top-down policy learning mechanism, cultivating the empathy of decision-makers and implementers, building a bottom-up interest expression mechanism, and fully utilizing the strength of college students to provide much-needed support for campus pandemic prevention and control. Finally, we anticipate that this study will help policymakers better grasp the needs of the policy target object, and offer solid empirical evidence for future policy improvement.

### Supplementary Information

Supplementary Information.

## Data Availability

The datasets generated and/or analysed during the current study are not publicly available due [restrictions by the Academic Committee of the School of Law, Shandong Jianzhu University to protect the participants’ privacy]. The datasets used and/or analysed during the current study available from the corresponding author (Caiyun Qi) on reasonable request.
