# Supplementary material for: Exploring the experiences of college students in Chinese campus lockdown policy during the COVID-19 outbreak: A qualitative study
Source: Sci Rep. 2023 Nov 14;13:19884. doi: 10.1038/s41598-023-47182-w (PMC10646024; doi:10.1038/s41598-023-47182-w)

**Supplementary Material**

﻿**Appendix 1. Word Cloud.** Highlighted topics and keywords from a variety of raw materials.


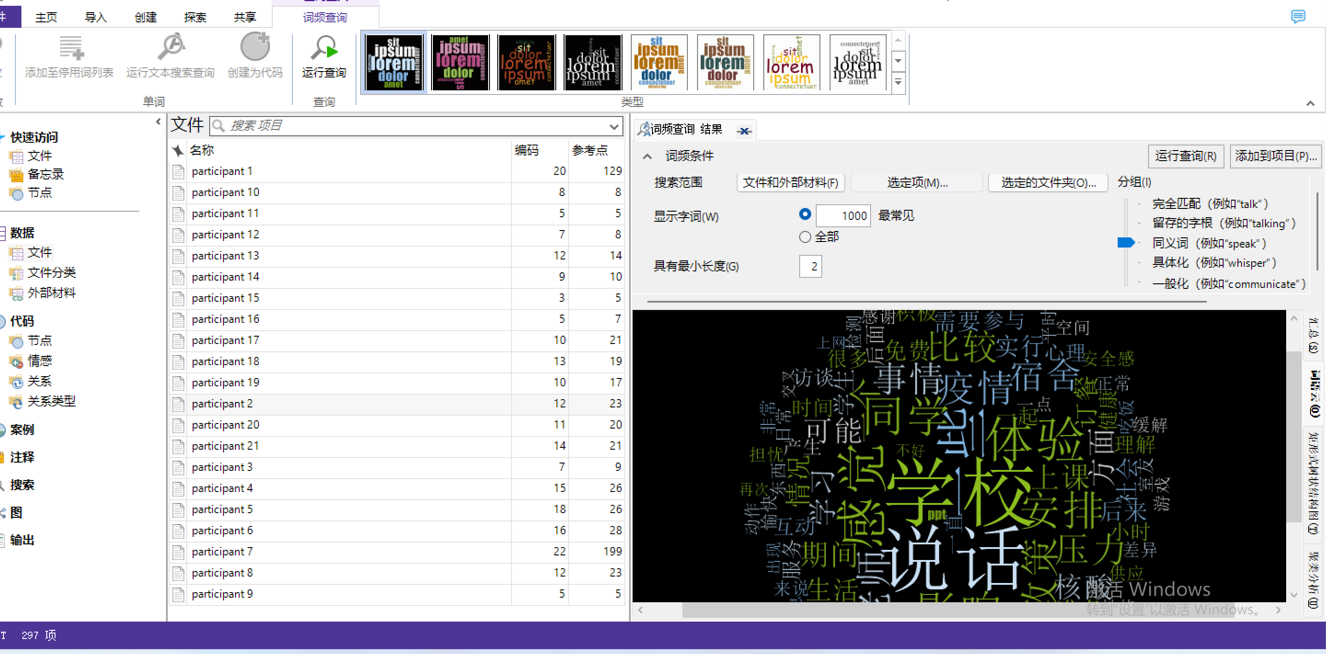


﻿

**Appendix 2. Coding and nodes.** Coding interview materials into themes.


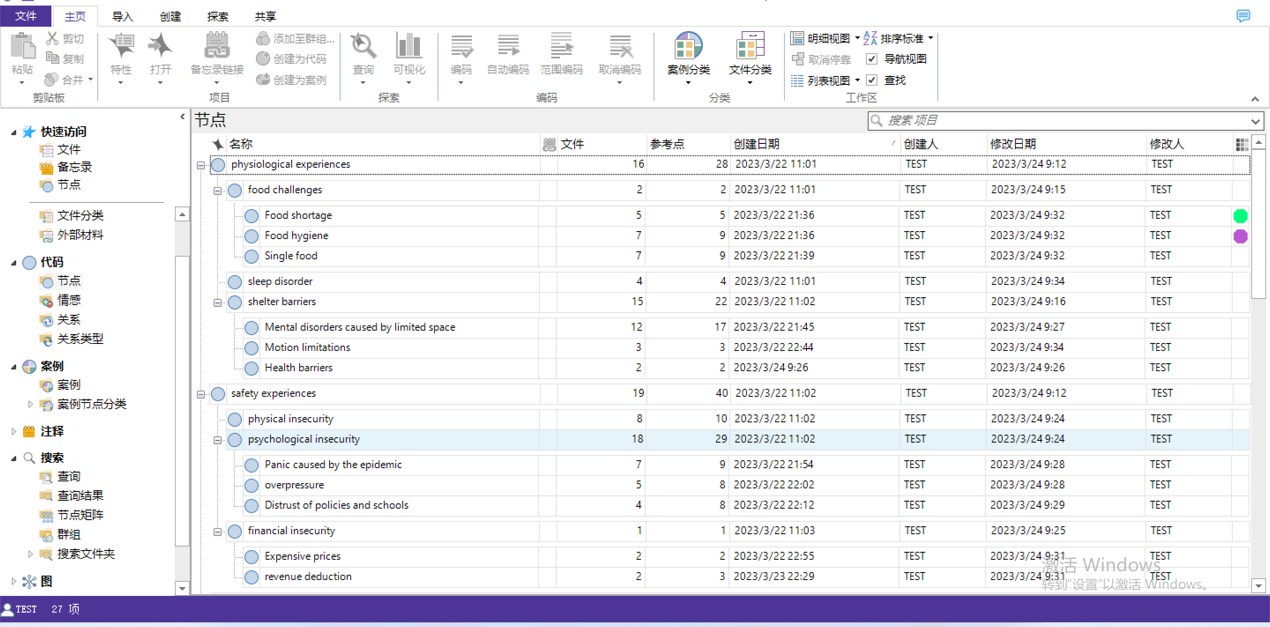


**Appendix 3. Coding comparison.** Comparison of interview material coding reference points.


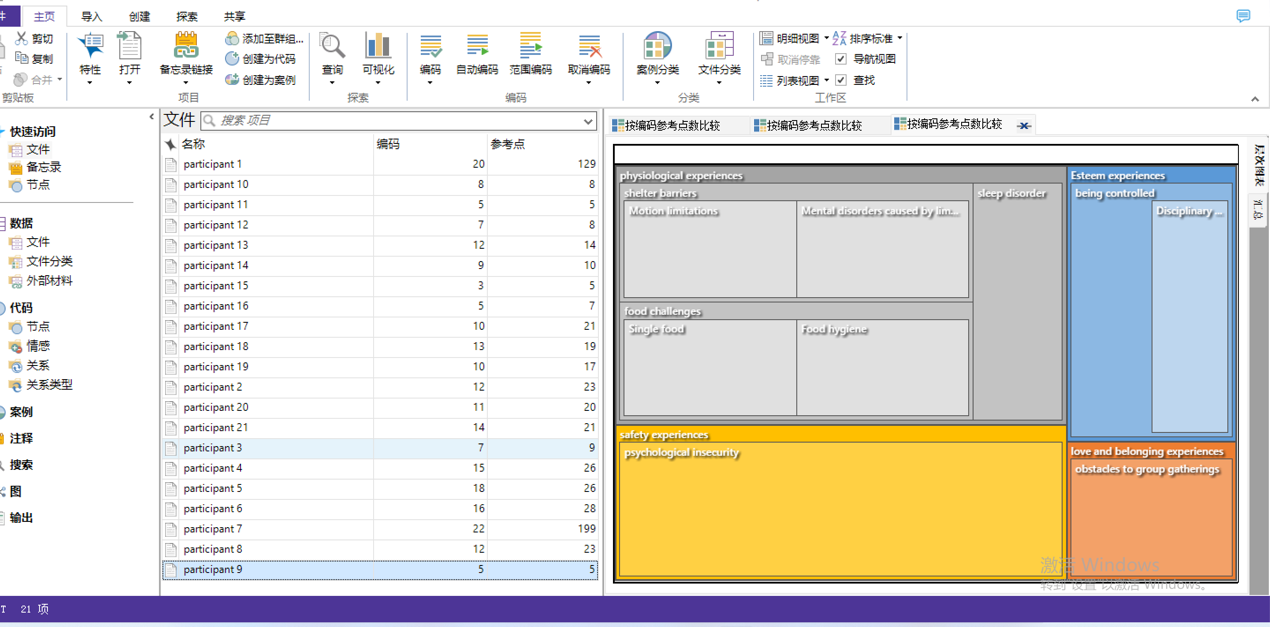


﻿

**Appendix 4. Coding stripes.** Code distribution in the global presentation.


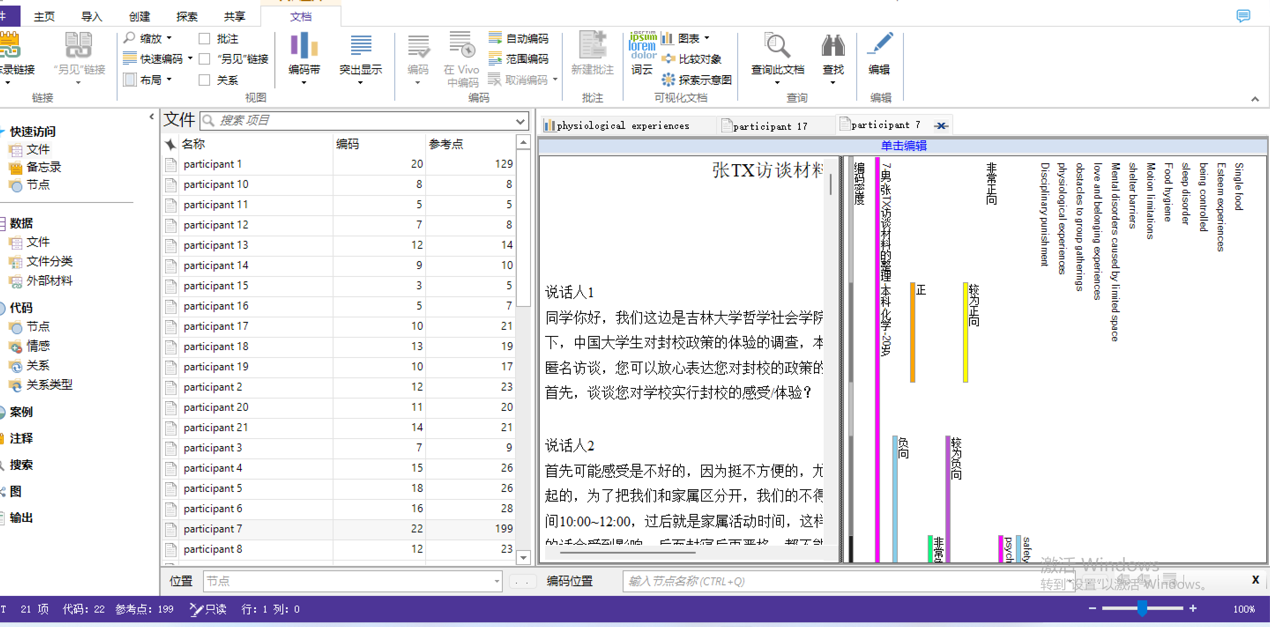


﻿

**Appendix 5. Coding by item.** Percentage of coverage per case encoded by the node.


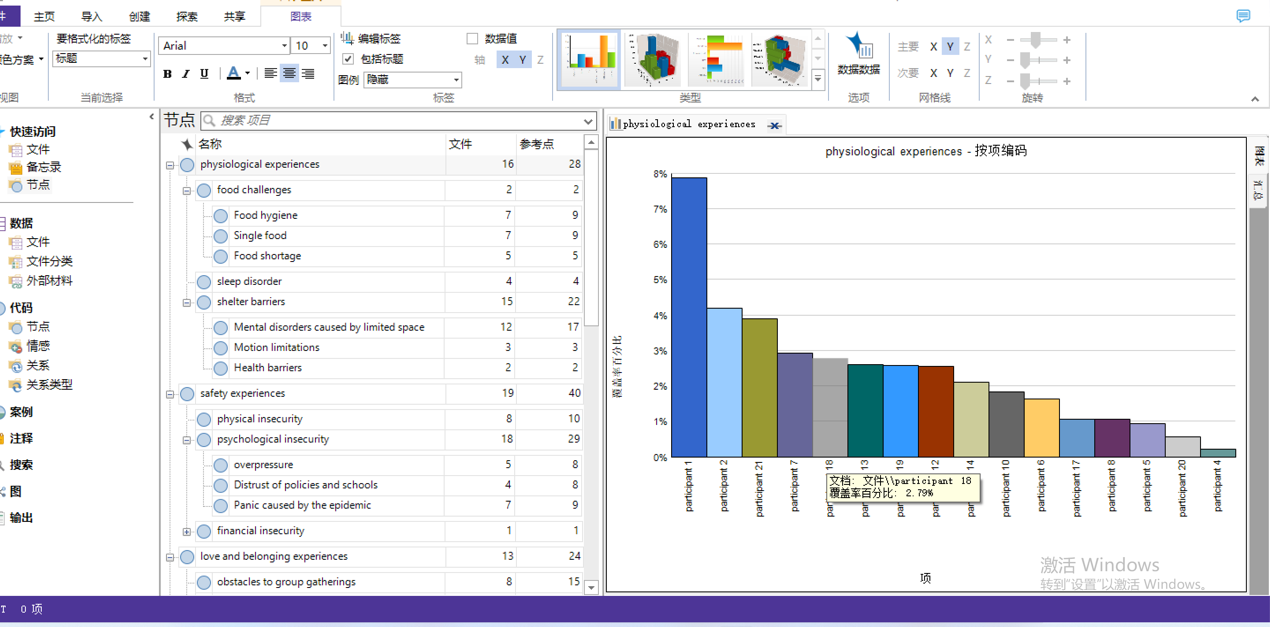

Supplement: Supplementary file 1 — Supplementary Information. [file 41598_2023_47182_MOESM1_ESM.doc]
